# Discovery of Antibodies Against Endemic Coronaviruses with NGS-Based Human Fab Phage Display Platform

**DOI:** 10.3390/antib14020028

**Published:** 2025-03-27

**Authors:** Oscar Chi-Chien Pan, Sean Miller, Ruchin Patel, Shreya Mukhopadhyay, Giancarlo Sarullo, Gwenny Go, Jennifer Galli, Jamie Hessels, Barbara Schlingmann-Molina, Emmanuel Ndashimye, Zhiyun Wen, Christopher Warren, Eberhard Durr, Lan Zhang, Kalpit A. Vora, Arthur Fridman, Zhifeng Chen

**Affiliations:** 1 Infectious Disease and Vaccines, Merck & Co., Inc., Rahway, NJ 07065, USA; chi-chien.pan@merck.com (O.C.-C.P.); jennifer_galli@merck.com (J.G.); eberhard.durr@merck.com (E.D.);; 2 Analytical R&D, Merck & Co., Inc., Rahway, NJ 07065, USA; sean.miller1@merck.com; 3 Eurofins PSS Insourcing Solutions, Lancaster, PA 17601, USA; 4 Quantitative Biosciences, Merck & Co., Inc., Rahway, NJ 07065, USA; 5 Data Science and Scientific Informatics, Research and Development Science-Information Technology, Merck & Co., Inc., Rahway, NJ 07065, USA

**Keywords:** monoclonal antibody, Human Fab phage display, PacBio, next-generation sequencing (NGS)

## Abstract

**Background:** There is an unmet medical need to develop a vaccine targeting endemic coronaviruses. Antigen-specific monoclonal antibodies (mAbs) are crucial for many assays to support vaccine development. **Objective:** In this study, we used the HuCal Fab phage display library with a diversity of 4.5 × 10^10^ to identify antibodies specific to the spike proteins of the four endemic coronaviruses: OC43, NL63, 229E, and HKU1. **Methods:** As proof of concept, we established a newly designed platform using a long-read NGS workflow for antibody discovery and compared the results against the traditional workflow using Sanger sequencing consisting of lengthy and laborious benchwork. **Results:** The long-read NGS workflow identified most of the antibodies seen from the Sanger sequencing workflow, and many more additional antigen-specific antibodies against the endemic coronaviruses. Overall efficiency improved up to three times, comparing the traditional workflow with the NGS workflow. Of the 113 NGS-derived mAbs isolated to bind the four endemic coronavirus spike proteins, 107/113 (94.7%) had potent ELISA binding affinities (EC50 < 150 ng/mL, or <1 nM), and 61/113 (54%) had extremely potent ELISA binding affinities (EC50 of <15 ng/mL, or <0.1 nM). **Conclusions:** We successfully developed and incorporated the long-read NGS workflow to generate target-specific antibodies with many antibodies at sub-nanomolar affinities that are likely missed by a traditional workflow. We identified strong neutralizing antibodies, proving that our endemic spike proteins are capable of generating antibodies that could offer protection against the endemic HCoVs.

## 1. Introduction

Coronaviruses are enveloped, positive-sense, single-stranded RNA viruses grouped into four genera: Alphacoronavirus, Betacoronavirus, Gammacoronavirus, and Deltacoronavirus [1]. There are seven coronaviruses from the Alpha and Beta genera that are known to cause infection and respiratory diseases in humans. Disease severity varies from common colds to severe acute respiratory syndrome (SARS). Due to the usually mild cold symptoms, coronaviruses were understudied prior to the SARS epidemic in 2003 [2]. It is estimated that the four endemic human coronaviruses (HCoV), HCoV-OC43, HCoV-229E, HCoV-NL63, and HCoV-HKU1, are responsible for up to one-third of the global common cold infections each year, causing a significant medical burden [3]. On the other hand, severe acute respiratory syndrome coronavirus (SARS-CoV), Middle East respiratory syndrome coronavirus (MERS-CoV), and SARS-CoV-2 are highly pathogenic and more likely to result in severe outcomes including death [4].

Coronaviruses contain unique surface projections that resemble spikes under an electron microscope [5]. The spike proteins on the coronavirus surface are critical for host cell infectivity, and they can be divided into two domains: the surface-exposed S1 domain and the membrane-proximal S2 domain. The receptor binding domain is located within the S1 portion of the spike protein [6]. The spike protein is highly immunogenic, and its receptor binding domain (RBD) is the major target for vaccines and neutralizing antibodies [7].

Monoclonal antibodies (mAb) against spike proteins are crucial for the development of assays to support endemic coronavirus vaccine development. A readily available tool for antibody discovery is through the panning of a large diversity phage display antibody fragment library. George P. Smith first developed phage display technology in 1985 [8], and the technology quickly advanced in the 1990s from peptide libraries [9] to recombinant antibody fragment libraries [10,11,12,13,14,15]. A major limitation of the traditional phage-panning platform is the laborious workflow and low throughput of obtaining antibody gene sequences relying on Sanger sequencing [16].

To improve upon the traditional workflow and obtain a more diverse panel of sequences after panning, a commonly used next-generation sequencing (NGS) platform is MiSeq from Illumina. Paired-end MiSeq (2 × 300 bp) sequencing from Illumina covers segments of about 500 bp and has been successfully applied to the investigation of shorter antibody fragment libraries, such as nanobodies derived from the camelid’s variable domain of heavy-chain-only antibodies [17,18], as well as the shark variable new antigen receptor (VNAR) [19]. Paired-end MiSeq can also read amplicons long enough to cover all CDRs in single-chain Fv (scFv) [20]. However, paired-end MiSeq is not sufficient to cover longer light and heavy chain-combined sequences (1.2–1.5 kb), which are linked together in Human Fab libraries [21,22].

Third-generation NGS technology such as Pacific Biosciences (PacBio) can bridge the shortcomings of current MiSeq sequencing [23], as it is possible to achieve long sequencing reads of 10 to 15 kb in length with single-molecule real-time technology (SMRT) [24]. PacBio’s NGS had been applied successfully to scFv fragment libraries [25,26]. In particular, Han et al. applied PacBio’s platform to the deep sequencing of the immune rhesus macaque scFv phage library to identify additional unique antibody gene sequences previously not seen in their Sanger sequencing results [26].

In this study, we used Bio-Rad’s HuCAL PLATINUM Human Fab phage library with a diversity greater than 45 billion. The HuCAL PLATINUM library is a second-generation, synthetic Human Fab library optimized from the previous HuCAL Gold library. The HuCAL PLATINUM library has been improved to contain less undesirable glycosylation while improving expression levels. The strategy for the design of its CDRs incorporated the length and distribution of natural amino acids in the human repertoire. Overall, the HuCAL PLATINUM library generates more unique sequences containing more high-affinity antibodies against targets when compared to the previous HuCAL Gold library [27].

Taking advantage of the deep sequencing power of the PacBio NGS platform, we developed a deep panning strategy using the HuCAL Platinum library to discover spike protein-specific antibodies for the endemic HCoV. We compared our NGS data set with the results from the traditional platform using Sanger sequencing. To the best of our knowledge, our study is the first to report applying PacBio long-read NGS in combination with the phage display panning of large-size Human Fab libraries.

## 2. Materials and Methods

### 2.1. Phage Display Library Panning

The procedure has been described previously [28]. The human combinatorial antibody HuCAL PLATINUM (Bio-Rad, Hercules, CA, USA) was used in cross-panning against OC43, NL63, 229E, HKU1-1, and HKU1-2 spike proteins (the HKU1 virus was categorized into HKU1-1 and HKU1-2 based on the differences in their spike proteins) produced in-house (Merck & Co., Inc., Rahway, NJ, USA). Briefly, all 7 kappa and 7 lambda antibody phage libraries were mixed together to increase the chance of identifying antigen-specific antibodies. To generate antibodies against spike proteins from OC43, NL63, 229E, HKU1-1, and HKU1-2, four rounds of cross-panning were conducted. To generate NL63-specific antibodies, we used OC43 as a negative selection before positive selection on NL63 and vice versa to obtain OC43-specific antibodies. The 229E and HKU 1-2 spike proteins both still retained their His tags. To eliminate antibodies against His tags, 229E was used as a negative selection for pooled HKU1-1/1-2 and vice versa. It should be noted that HKU1-1 and 1-2 were pooled because we were interested in finding antibodies specific to each spike protein as well as cross-reactive antibodies to both HKU1-1 and 1-2. After extensive washing, phages binding to the spike protein were eluted with 20 mM DTT in 10 mM Tris/HCl pH 8.0 and amplified by infection and the overnight culture of TG1 F+ *E. coli*. The amplified phages were harvested and used in the second round of cross-panning with negative and positive selections. Subsequent rounds of panning followed the same strategy and protocol.

### 2.2. Subcloning, Screening, Sanger Sequencing, and Identification of Antigen-Specific Bivalent Fabs Using Traditional Approach

This traditional approach with Sanger sequencing has been described previously [28]. After four rounds of cross-panning, the *E. coli* colonies grown on LB agar plates with 1% glucose and 34 μg/mL chloramphenicol (Teknova, Hollister, CA, USA) were scraped off and resuspended in 2 × YT broth (Teknova) with chloramphenicol. The bacterial pellets were harvested for plasmid DNA preparation with the QIAprep Spin Miniprep Kit (Qiagen, Germantown, MD, USA). Minipreps of plasmid DNA were digested with EcoR I and Xba I, and the 1.4 kb DNA fragments encoding antibody sequences were agarose-gel-purified with the QIAquick Gel Extraction kit (Qiagen) and subcloned into a bivalent Fab expression vector pMORPH × 9_Fab_dHLX_MH with the Myc tag and His6 tags (Bio-Rad).

Constructs with antibody DNA fragment inserts were used to transform *E. coli* Stellar competent cells (Mountain View, CA, USA), which were then spread onto LB agar square plates with 1% glucose and 34 μg/mL chloramphenicol (Teknova). The plates were incubated at 37 °C overnight. Each transformant was grown in 100 μL of 2 × YT broth containing 1% glucose and 34 μg/mL chloramphenicol in a master 96-well plate at 37 °C overnight, shaking at 250 rpm. In total, 5 μL of overnight culture from each well of the master plate was inoculated into a 96-well expression plate containing 100 μL of 2 × YT broth with 34 μg/mL of chloramphenicol per well. The expression plate was incubated at 37 °C with shaking for 4–6 hrs. The bivalent Fab expression was induced by the addition of 20 μL per well of 2 × YT broth with 34 μg/mL chloramphenicol and 3 mM IPTG (at a final concentration of 0.5 mM IPTG). The expression plate was cultured overnight at 30 °C with shaking.

Overnight-induced *E. coli* cultures were first frozen at −70 °C for 1 h, then thawed under room temperature. Bacteria were then lysed by the addition of 40 μL per well of the BEL buffer (400 mM Boric Acid, 300 mM NaCl, 5 mM EDTA, and 2.5 mg/mL Lysozyme) and shaking at 400 rpm for 1 h at room temperature. In total, 40 μL of 12.5% non-fat dry milk prepared in 1 × TBS (Fisher Scientific, Waltham, MA, USA) was added and incubated for 30 min at room temperature with shaking at 400 rpm. Samples were tested without dilution in the ELISA assay described below for binding to respective spike proteins. Wells with fluorescence signals 2-fold higher than the negative control (no Fab lysate) were treated as positive hits and picked for Sanger sequence analysis. Unique antibody sequences were identified, and corresponding bivalent Fabs were then produced if the sequence appeared three times or more.

### 2.3. Long Read NGS

The gel-purified 1.4 kb DNA fragments sent to Azenta (Azenta, Burlington, MA, USA) for long-read NGS are the same DNA fragments used for subcloning into the bivalent Fab expression vector described earlier. Briefly, to prepare the SMRTbell library for PacBio Sequel (Pacific Biosciences, Menlo Park, CA, USA), the DNA samples were treated for DNA damage repair and A-tailing reactions. The samples were ligated with PacBio’s SMRTbell overhang adaptors to create complete, intact SMRTbell templates. Indexed SMRTbell adaptors were used when multiplexing multiple samples. SMRTbell cleanup beads were used to concentrate and purify prepared SMRTbell library DNA. SMRTbell libraries were loaded onto PacBio Sequel I. CCS reads were analyzed with in-house (Merck & Co., Inc., Rahway, NJ, USA) analysis tools to extract antibody-heavy and light-chain CDR 1, 2, and 3 sequences.

### 2.4. Conversion and Production of Full-Length Human IgG1

The procedure has been described previously [28]. Heavy- and light-chain variable region sequences were subcloned into expression vectors encoding human IgG1 constant regions for the transient co-transfection of CHO-Express cells, and purified antibodies were obtained. The purified antibodies were buffer-exchanged to 1 × PBS and QC-tested by SDS-PAGE and Western blot analysis. (GenScript, Piscataway, NJ, USA).

### 2.5. ELISA

The procedure has been described previously [28]. To test bivalent Fabs, 96-well Maxisorp ELISA plates (Thermo Scientific, Waltham, MA, USA) were coated with respective spike proteins overnight at 4 °C. Three-fold serially diluted antibodies were prepared in 1% nonfat milk/TBST, transferred to antigen-coated plates, and incubated for 1 h at room temperature. The plates were washed 6 times with PBST. In total, 100 μL per well of 1:10,000-diluted alkaline phosphatase-conjugated goat anti-human IgG F(ab′)2 (Jackson ImmunoResearch, West Grove, PA, USA) was then added and incubated for 1 h at room temperature. Plates were washed 6 times with PBST. In total, 100 μL of the AttoPhos substrate (Promega, Madison, WI, USA), diluted 1:5 in TBST, was added to each well. After 4–7 min of incubation at room temperature, fluorescence signals were read with a Tecan F500 plate reader at 435 nm for excitation and 530 nm for emission. To test full-length human IgGs, six-fold dilutions of antibodies were used and detected with 1:10,000-diluted alkaline phosphatase-conjugated goat anti-human IgG Fc (Jackson ImmunoResearch), which was used as a secondary antibody. Data were analyzed with GraphPad Prism (version 10.2.2, San Diego, CA, USA).

### 2.6. Pseudovirus Neutralization Plaque Assay

For NL63, 229E, HKU1-1, HKU1-2, SARS-CoV, SARS-CoV2, and MERS, the following protocol was used. Briefly, mAb samples were diluted in black, clear-bottom, 384-well plates (Corning, Corning, NY, USA) containing DMEM with 2% FBS (Thermo Fisher Scientific). We added in-house-generated (Merck & Co., Inc., Rahway, NJ, USA) pseudovirus engineered with GFP expression, which was incubated for 1 h at 37 °C, 5% CO_2_, and 80% relative humidity. We added 786-O cells (ATCC, Gaithersburg, MD, USA) for 229E and MERS pseudoviruses; for NL63, HKU1-1, and HKU1-2 pseudoviruses, we added HEK Blue cells (Invivogen, San Diego, CA, USA); and for SARS-CoV and SARS-CoV2 pseudoviruses, we added A549 cells (GenScript) to each well and incubated them for 72 h at 37 °C, 5% CO_2_, and 80% relative humidity. We read the GFP foci count with an Acumen HCS reader (SPT Labtech, Melbourne, UK) at 488 nm. For OC43, antibodies were diluted in black, clear-bottom, 96-well plates. HRT-18 cells were added and then incubated for 72 h at 37 °C, 5% CO_2_. A GFP signal read was obtained with Acumen Cellista (SPT Labtech). Data were analyzed with GraphPad Prism (version 10.2.2).

### 2.7. Wild-Type Virus Neutralization Plaque Assay

For OC43 and NL63, the following protocol was used. Briefly, mAb samples were diluted in black, clear-bottom, 96-well plates. The OC43 or NL63 wild-type virus (BEI Resources, Manassas, VA, USA) at around 100 pfu/well was added to mAb samples and incubated for 1 h at 37 °C, 5% CO_2_. Appropriate cells were added and then incubated with layered methylcellulose for 72 h at 37 °C, 5% CO_2_. Cells were fixed with formaldehyde, and plaques were developed with anti-OC43 or anti-NL63 antibodies stained with Alexa 488-conjugated secondary antibodies. Plates were read with the EnSight Multimode Plate Reader (Revvity Health Sciences Inc., Waltham, MA, USA). Data were analyzed with GraphPad Prism (version 10.2.2).

### 2.8. Biolayer Interferometry Binding Affinity Measurements

Biolayer interferometry (BLI) assays were performed using the default kinetics measurement protocol on an Octet Red 96 instrument (Sartorius, Bohemia, NY, USA). A baseline was established before and after the loading of biotin-labeled spike proteins by immersing them into wells containing a 1 × Octet kinetics buffer (Sartorius). Spike proteins (3 μg/mL) conjugated with EZ-Link NHS-peg4-biotin (Thermo Fisher Scientific) were immobilized onto streptavidin biosensors (Sartorius). Finally, the antibodies at various concentrations starting from 100 nM in the 1 × Octet kinetics buffer were associated for 5 min and dissociated for 5 min in wells with a 1 × Octet kinetics buffer. After each run, the results were analyzed with instrument software using global fit and a 2:1 heterogeneous model. Data points were graphed using GraphPad Prism (version 10.2.2) for clarity.

## 3. Results

### 3.1. Traditional Phage-Panning Workflow vs. Long-Read NGS Workflow

**Figure 1:**
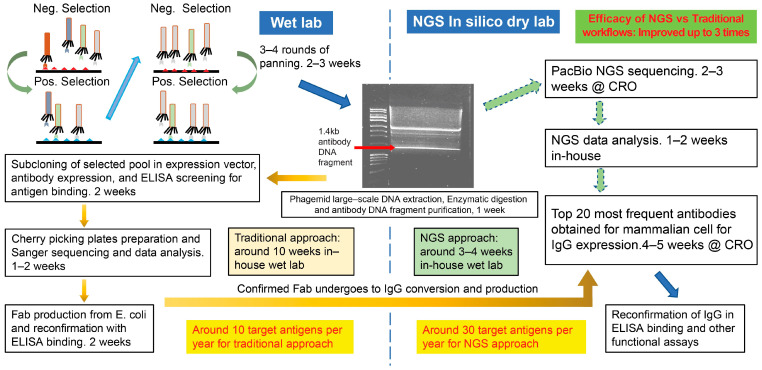
Comparison of traditional and NGS phage display workflows. After four rounds of panning, phagemid DNA was purified and digested with restriction enzymes. The 1.4 kb antibody gene DNA fragment is the common starting point for traditional and NGS workflows. The traditional workflow involves cloning and expressing antibody genes as bivalent Fab for the ELISA test. Clones with positive and specific antigen binding are cherry-picked for Sanger sequencing. The most frequent antibody sequences are scaled up as bivalent Fab for further ELISA characterization before human IgG conversion and production. In contrast, in the NGS workflow, the antibody gene DNA fragment is directly sent out for PacBio sequencing. The top 20 most frequent antibody sequences from the NGS results are converted into human IgG and production.

In the traditional workflow (Figure 1) using Sanger sequencing, the antibody gene fragments digested from phagemid DNA were cloned into expression vectors for bivalent Fab. The expression vectors transformed *E. coli*, and the cell lysate was assessed for specific antigen binding by ELISA. The positive binding colonies were cherry-picked for Sanger sequencing. Antibody sequences appearing three times or more in Sanger sequencing were expressed in larger-scale production as bivalent Fab in *E. coli* for further binding characterizations before their conversion into full-length human IgG1.

For the NGS workflow (Figure 1), the same antibody gene fragment digested from phagemid DNA was directly sent out for PacBio long-read NGS. The top 20 or more of the most frequent antibodies observed in the NGS data sets were directly expressed as full-length human IgG1 for testing. Due to the substantially less hands-on bench work time involved in the NGS workflow, we were able to focus on panning for the next set of target antigens, thus improving our throughput by three times per year for the number of target antigens screened for antibody generation.

### 3.2. Antibody Gene Sequences Identified Through Traditional vs. NGS Platforms

For antibody frequency determination, the antibody gene sequences were organized by heavy-chain complementary-determining region 3 (HCDR3). All HCDR3 sequences identified from the traditional workflow that appeared more than twice are listed next to their frequency rank and NGS frequency rank (Table 1). The side-by-side traditional vs. NGS antibody frequency rankings indicate that most of the frequent antibodies identified in the traditional workflow were also observed in the top 20 most frequent antibodies by the NGS workflow. As can be observed in the gaps in the NGS frequency ranking compared to the traditional workflow, all the antibodies ranked in the NGS frequency gaps were additional antibodies not seen in the traditional workflow. Thus, the NGS workflow identified thousands of antibody sequences that were likely not found using the traditional workflow. Due to the smaller sample size in the Sanger sequencing of 96 samples, the antibody sequence frequency ranking varied significantly for antibody sequences other than the dominant top one or two clones when compared to the NGS frequency ranking. The most frequent antibody sequences were not so apparent for HKU1-1/1-2 panning since these two spike proteins were pooled during panning. As the NGS platform used in this study was PacBio, all the NGS-ranked antibodies (shown in Table 1 below) were named PACB1, PACB2, etc.

### 3.3. Antibody Binding Confirmation ELISA Against Their Respective Antigens

**Figure 2:**
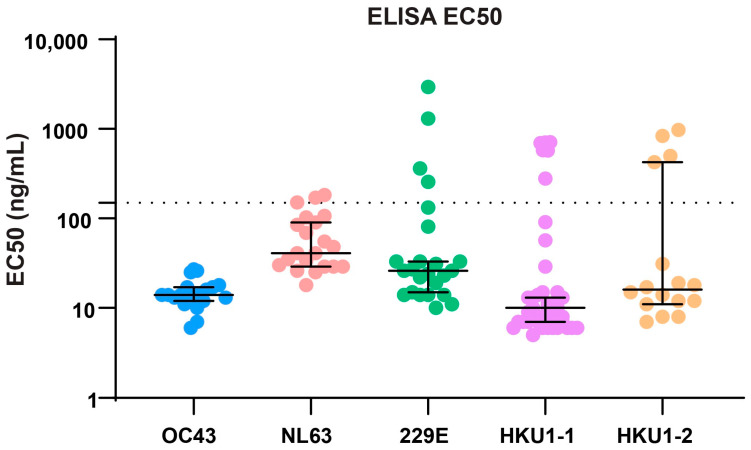
ELISA EC50 values of all expressed antibodies. Horizontal lines represent median with 95% confidence intervals shown. All data points below dotted line have EC50 values less than 150 ng/mL (1 nM).

Antibodies identified from the traditional workflow were already shown to bind to their respective antigen in screening ELISA prior to expression as full-length human IgG1 antibodies. The most frequent antibody sequences identified by NGS workflow were not verified by ELISA to show positive antigen binding prior to their expression as full-length human IgG1 antibodies, to save time and labor. ELISA was performed on all expressed antibodies to verify they bind to their respective antigens. The ELISA binding results showed 100% positive binding from all top frequency antibodies from NGS workflow against their respective spike protein targets (Figure S1 and Figure 2, Table 2). The ELISA 50% Effective Concentration (EC50) values were similar among most frequent antibodies vs. the less frequent antibodies as can be seen from the antibody name that contains NGS frequency rank.

Over 94% of antibodies generated had ELISA EC50 values (Figure 2) less than 150 ng/mL (<1 nM); furthermore, over 54% of the antibodies had EC50 values less than 15 ng/mL (<0.1 nM). Table 2 summarized the median EC50 values and range of EC50 values analyzed. All antibodies were tested for binding against their negative selection panning antigen, results indicated all antibodies were specific against their respective positive selection antigen. In the generation of HKU antibodies, both HKU1-1 and HKU1-2 were pooled during panning since we wanted antibodies specific to each spike protein and also antibodies that cross-reacted to both HKU spike proteins. Results showed we successfully generated specific antibodies against HKU 1-1 and 1-2 spike proteins, respectively. Additionally, we also found antibodies that were specific or cross-reactive to both HKU1-1 and HKU1-2.

### 3.4. Pseudovirus Neutralization

**Figure 3:**
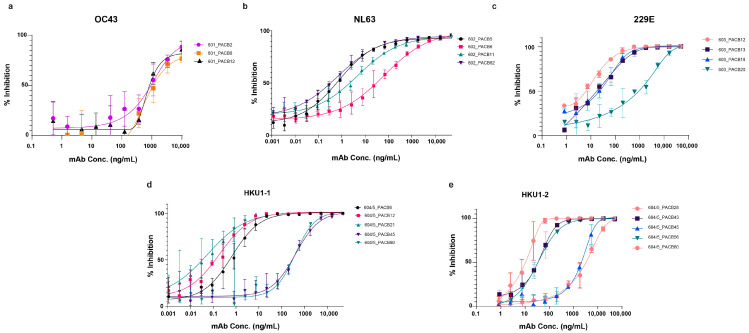
Pseudovirus neutralization curves for top-neutralizing mAbs for OC43 (**a**), NL63 (**b**), 229E (**c**), HKU1-1 (**d**), and HKU1-2 (**e**). Antibodies were serially titrated and incubated with their respective pseudovirus for 1 h at 37 °C before adding cells and incubating for 72 h at 37 °C. Antibodies were tested against different pseudoviruses, and none of the antibodies showed cross-neutralization. For example, NL63-neutralizing antibodies did not show neutralizing activity on OC43 pseudovirus and were considered a negative control in this OC43 neutralization assay. The same conditions were applied to other neutralizing antibodies.

The pseudovirus neutralization assay was performed on all antibodies against their corresponding GFP-expressing pseudovirus. Neutralizing antibodies were identified against each of the respective spike proteins. The 50% inhibition values ranged from less than 1 ng/mL to about 1 ug/mL (Figure 3, Table 3). The HKU1-1 and HKU1-2 cross-reactive antibodies did not neutralize either HKU1-1 or HKU1-2 as potently as HKU1-1-specific or HKU1-2-specific antibodies (Table 4). All neutralizing antibodies were evaluated for cross-neutralization against other endemic HCoVs as well as SARS-CoV, SARS-CoV2, and MERS. As expected, our endemic HCoV-neutralizing antibodies did not show any cross-neutralization to any other coronaviruses.

### 3.5. Wild-Type Virus Neutralization

**Figure 4:**
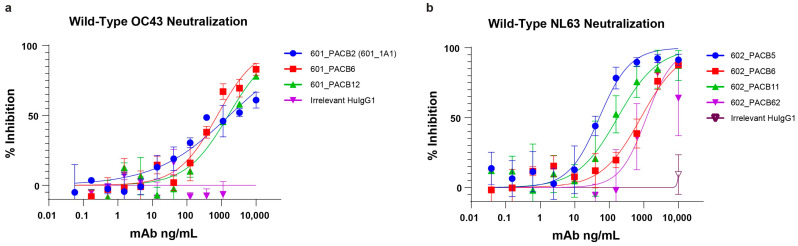
Wild-type NL63 and OC43 HCoV neutralization. Previously found OC43 or NL63 neutralizing antibodies were serially titrated in duplicate and incubated with an appropriate wild-type virus. After 1 h at 37 °C, cells were added and incubated for 72 h at 37 °C. Cells were fixed with formaldehyde, and plaques were developed with anti-OC43 or anti-NL63 antibodies stained with Alexa 488-conjugated secondary antibodies. (**a**) Wild-type OC43 HCoV neutralization; (**b**) wild-type NL63 HCoV neutralization.

To test whether the pseudovirus-neutralizing mAbs were capable of neutralizing wild-type viruses, we tested OC43- and NL63-neutralizing mAbs for wild-type virus neutralization. Our results (Figure 4) showed that all of the OC43 pseudovirus-neutralizing mAbs were capable of neutralizing the wild-type OC43 virus. Similarly, all of the NL63 pseudovirus-neutralizing mAbs exhibited neutralizing activity towards the wild-type NL63 virus. The IC50 values of all tested antibodies were less than 1 ug/mL against either the OC43 or NL63 wild-type virus. All IC50 values are summarized in Table 3.

### 3.6. Biolayer Interferometry (BLI) Affinity Assessment

To obtain a more accurate assessment of binding affinity to back up the ELISA binding results, we used a biotinylated version of the respective spike proteins captured on streptavidin biosensors and measured the binding of the neutralizing mAbs through BLI. The experiment used spike proteins, which are homotrimers of the S protein, and full-length antibodies with bivalent antigen binding sites. One antigen binding site on the antibody molecule could interact with one site on one S protein of the trimer spike protein. It is also possible for both antigen binding sites of an antibody to simultaneously bind to two of the three S proteins in the trimer. Therefore, the association and dissociation curves were analyzed with the 2:1 heterogeneous model (Figure S2). The BLI kinetic measurement conditions were set at 5 min for the association phase and 5 min for the dissociation phase for the affinity assessment of the antibodies. To properly determine the dissociation constant, one-to-one interactions between the antigen and antibody should be considered using longer association and dissociation times. One-to-one interactions may be possible with very diluted antibody and/or antigen concentrations, but the signals would be much weaker, and accurate analysis may not be possible with lower readings.

The apparent K_D_ values obtained from BLI (Table 3 and Table 4) indicated that most neutralizing antibodies for the respective endemic HCoV were sub-nanomolar in affinity. Some binding curves exhibited fast association and fast dissociation within the first minute of each step. During dissociation, the curves quickly leveled off after the initial quick release of some of the antibodies. All 229E antibodies (Figure S2c) had relatively flat dissociation curves without the characteristic sharp drop-off in the first minute of the dissociation step, like most of the other antibodies tested. All HKU1-1 antibodies (Figure S2d) had 10 min for association and dissociation. Three out of the five HKU1-1 antibodies had apparent K_D_ estimates out of range at <1 × 10^−12^ M.

### 3.7. Neutralizing Antibody Germline Sequence Analysis

The initial HuCAL phage library does not have a bias for any particular germline antibody sequence [27]. The germline sequence of our pseudovirus-neutralizing antibodies showed preference towards VH 1-69 for heavy-chain and VK 1-39 for light-chain (Table 2 and Table 3). Table 1 shows the germline usage of all expressed mAbs with high frequency using NGS against the spike proteins of endemic coronaviruses.

## 4. Discussion

The newly developed NGS workflow is extremely beneficial in the rapid advancement of monoclonal antibody discovery. Direct costs for sequencing through Sanger or PacBio platforms are similar, but the indirect costs (bench scientist time, etc.) are much lower with the NGS platform. The incorporation of the NGS workflow bypassed weeks of hands-on bench work. We were able to start panning immediately for the next set of antigen targets when the first batch was in the NGS sequencing stage. The improved efficiency translated into a three-fold increase in the number of target antigens that we can process per year. Furthermore, the PacBio NGS platform’s long-read capability provided us with tens of thousands of sequencing reads, which is impossible to achieve with a traditional workflow using Sanger sequencing. With the power of deep sequencing and deep mining of antibody sequences, we generated many antibodies that would have been likely to be missed by the traditional workflow. Furthermore, with a much larger data set sequence, the NGS approach provided a more accurate antibody frequency ranking, as can be seen in Table 1.

From the initial 4.5 × 10^10^ phage library diversity and over 1 × 10^12^ phage particles input for panning, we obtained highly specific antibodies against each of the endemic coronaviruses’ spike proteins with high affinity. The output phage at the end of four rounds of panning endured a total of over 60 stringent washes, which guarantees the efficient removal of non-specific binding phages. It is unsurprising that all the tested antibodies identified from the NGS workflow were specific and had high affinity even without the previous ELISA screening used in the traditional workflow.

The numbering in the antibody ID reflects its frequency ranking among NGS-identified antibodies. There is no correlation between antibody rank and binding strength. In fact, most antibodies with a lower frequency ranking had similar ELISA titration curves compared to higher-ranking antibodies. As seen in Table 3, none of the 229E-neutralizing antibodies contained a second ID from the traditional workflow, indicating that all these neutralizing antibodies were identified from the NGS workflow. This is a great example of where the power of NGS was able to identify many additional antibodies, in this case, 229E-neutralizing antibodies, which will very likely be missed in the traditional workflow unless further screening is carried out. In addition to all the previously discussed benefits of the NGS workflow, our results show the depth and quality of the antibodies identified, which greatly improves antibody discovery efforts and outcomes by providing many more valuable potential candidates.

Most of the neutralizing antibodies had less than 15 ng/mL (0.1 nM) IC 50 values for their respective pseudovirus neutralization assay. We showed that the OC43 and NL63 pseudovirus neutralizers were also capable of neutralizing their respective wild-type OC43 and NL63 viruses. None of the pseudovirus-neutralizing antibodies showed cross-neutralization against other endemic HCoVs and other coronaviruses such as SARS-CoV, MERS, or SARS-CoV2. This outcome was expected since our phage-panning strategy was focused on finding antigen-specific antibodies. To identify cross-reactive antibodies, the panning strategy will need to be modified for a more inclusive screening selection of all desired target antigens during panning.

The ELISA EC50 values indicated that the majority of the additional antibodies generated through the NGS workflow had strong binding to their respective antigens. BLI affinity measurement conditions with the trimeric antigen and bivalent antibodies represented a similar protein interaction setup to ELISA. BLI affinity data confirmed strong sub-nanomolar affinities for almost all neutralizing antibodies. The ELISA EC50 values and the BLI apparent for K_D_ for the HKU1-1 and HKU1-2 cross-reactive and cross-neutralizing antibodies were in very good agreement. Although BLI affinity was not performed for the rest of the antibodies, good data agreement between ELISA EC50 values and BLI affinity estimates for the neutralizing antibodies extended our confidence in the ELISA EC50 values for all the antibodies tested.

Antibody gene analysis from all 113 antibodies showed a strong preference for VH1-69 (50/113) and VK1-39 (50/113) (Figure S3). Of the top 19 neutralizing antibodies, 11 antibodies used VK1-39, and 7 antibodies used VH1-69 (Table 2 and Table 3). The composition of the HuCAL PLATINUM library was not biased towards VK1-39 and VH1-69 [27]. The nature of our spike protein targets and the panning selection strategy were preferentially enriched for VK1-39 and VH1-69, especially for NL63 mAbs (16/21 for both VK1-39 and VH1-69 usage). We suspect that the specific germline usage of VH1-69 and VK1-39 is relevant for the better fitting of antibody binding to antigens. Structural studies are warranted to investigate this hypothesis. Indeed, the VH1-69 germline is often found in a panel of antibodies against infectious agents, such as the hemagglutinin of influenza, the E2 antigenic region 3 in HCV, and the CD4 binding site on GP120 in HIV [29]. Weber et al. took into consideration mutations within CDRH1, 2, and 3 to create a de novo fully synthetic antibody with VH1-69 that was able to neutralize various HCV genotypes [30]. The higher frequency of VH1-69 antibodies discovered in this study is in line with observations from the above investigators. Besides the PacBio NGS platform, there is a recent report about using Oxford Nanopore Technology (ONT) to analyze phage display data [31,32]. We are actively investigating this emerging new NGS technology.

In conclusion, we successfully developed and incorporated the long-read NGS workflow to generate target-specific antibodies with many antibodies at sub-nanomolar affinities that are likely missed by a traditional workflow. We identified strong neutralizing antibodies, proving that our endemic spike proteins are capable of generating antibodies that could offer protection against the endemic HCoVs. Our newly developed workflow with long-read NGS can be extended to other large DNA fragment library platforms and increase their throughput greatly.

## Figures and Tables

**Table 1:** Antibody heavy-chain CDR3 sequence frequency rankings for the traditional (Sanger) and NGS workflows (antibody heavy-chain and light-chain germline are also shown). The ranking for NGS is the same as in PACB nomenclature.

OC43 mAbs				HKU1-1/1-2 mAbs			
NGS Rank	Sanger Rank	VH Germline	VL Germline	NGS Rank	Sanger Rank	VH Germline	VL Germline
1	1	IGHV3-23	IGKV1S17	1	7	IGHV3-23	IGLV3-1
2	2	IGHV1-69	IGKV1-39	2	2	IGHV1-69	IGKV1-39
3	7	IGHV1-69	IGKV1-12	3	1	IGHV3-15	IGLV2-14
4	4	IGHV1-2	IGLV3-1	4	4	IGHV3-23	IGKV1-16
5		IGHV1-69	IGKV1-39	5		IGHV3-23	IGLV3-1
6		IGHV1-2	IGKV1-39	6	2	IGHV3-23	IGLV3-1
7	4	IGHV1-69	IGLV3-1	7	7	IGHV1-69	IGLV3-1
8		IGHV3-23	IGKV1S22	8	11	IGHV1-69	IGKV3-11
9	7	IGHV1-69	IGKV1-39	8	11	IGHV1-69	IGKV1S8
10		IGHV3-23	IGLV1-44	9	11	IGHV1-2	IGKV3-11
11		IGHV1-69	IGKV1-39	10		IGHV1-2	IGKV1-39
12	9	IGHV3-23	IGLV1-8	11		IGHV5-10-1	IGKV1S8
13	3	IGHV1-69	IGLV2-14	12	11	IGHV1-2	IGKV1-39
14	9	IGHV3-23	IGLV3-1	13	11	IGHV1-69	IGKV1-39
15		IGHV1-69	IGKV1-39	14		IGHV1-2	IGKV1-39
16	9	IGHV1-69	IGKV1-39	15	4	IGHV3-23	IGKV3-11
17		IGHV3-23	IGKV2-28	16	11	IGHV3-23	IGLV2-14
18	9	IGHV3-23	IGKV1-39	17	11	IGHV3-23	IGLV1-47
19	4	IGHV3-23	IGKV3-11	18	11	IGHV3-15	IGLV1-40
20	9	IGHV1-69	IGKV1-39	19	10	IGHV1-69	IGKV1-39
				20	11	IGHV3-15	IGLV3-1
NL63 mAbs				21	11	IGHV1-2	IGKV3-11
NGS Rank	Sanger Rank	VH germline	VL germline	22		IGHV3-23	IGLV2-14
1	1	IGHV1-69	IGKV1-39	23		IGHV3-15	IGLV3-1
2	16	IGHV1-69	IGKV1S8	24		IGHV1-69	IGKV1-39
3		IGHV1-69	IGKV1-39	25		IGHV3-23	IGLV1-44
4		IGHV1-69	IGKV1-39	26	11	IGHV3-23	IGLV1-40
5	2	IGHV5-51	IGLV1-47	27	11	IGHV1-69	IGKV1-39
6	10	IGHV3-23	IGKV1-39	28	10	IGHV5-10-1	IGKV1-39
7		IGHV1-69	IGKV1-39	29	11	IGHV3-15	IGLV3-1
8	16	IGHV1-69	IGKV1-39	30		IGHV3-15	IGLV3-1
9		IGHV1-69	IGKV1-39	35	11	IGHV1-2	IGLV3-1
10		IGHV1-69	IGKV1-39	36	11	IGHV1-69	IGKV1-39
11		IGHV1-69	IGKV1-39	37	11	IGHV3-15	IGKV1-39
12		IGHV1-69	IGLV3-1	41	11	IGHV1-69	IGKV1-39
13	16	IGHV1-2	IGKV1-16	43	4	IGHV6-1	IGKV1-39
14	6	IGHV1-69	IGKV1-39	45	11	IGHV1-69	IGKV1-12
15		IGHV1-69	IGKV1-39	49	11	IGHV3-23	IGLV3-1
16		IGHV1-69	IGKV1-39	51	11	IGHV3-15	IGLV3-1
17	6	IGHV1-2	IGLV3-1	56	11	IGHV5-51	IGKV1-39
18		IGHV1-69	IGKV1-39	58	7	IGHV3-23	IGKV1-39
19	16	IGHV1-69	IGKV1-39	67	11	IGHV1-69	IGLV1-40
20	3	IGHV3-23	IGLV3-1	71	11	IGHV3-23	IGLV3-1
62	3	IGHV1-69	IGKV3-11	80	11	IGHV1-69	IGKV3-11
				84	11	IGHV1-69	IGLV3-1
229E mAbs				93	11	IGHV3-15	IGLV1-40
NGS Rank	Sanger Rank	VH germline	VL germline	97	11	IGHV3-23	IGKV2-28
1	1	IGHV3-23	IGKV3-11	99	11	IGHV3-15	IGLV3-1
2	2	IGHV5-51	IGKV1-39				
3		IGHV1-69	IGKV1-39				
4	5	IGHV3-15	IGKV3-11				
5		IGHV3-15	IGLV1-44				
6	8	IGHV3-15	IGLV2-14				
7		IGHV1-69	IGKV1-39				
8	4	IGHV1-69	IGKV1-39				
9		IGHV3-15	IGLV2-14				
10	3	IGHV1-69	IGKV1-39				
11		IGHV1-69	IGKV1-39				
12		IGHV1-69	IGKV1-39				
13		IGHV6-1	IGKV1-39				
14		IGHV3-15	IGLV2-14				
15		IGHV1-69	IGKV1-16				
16		IGHV2-70	IGLV3-1				
17	5	IGHV3-15	IGKV1-39				
18		IGHV3-23	IGLV1-8				
19		IGHV3-15	IGLV3-1				
20		IGHV1-69	IGKV3-11				
23	8	IGHV1-69	IGKV1-39				
37	5	IGHV3-23	IGKV1-39				
66	10	IGHV1-69	IGKV1-39				
77	10	IGHV3-15	IGLV3-1				

**Table 2:** Antibody EC50 values.

	OC43	NL63	229E	HKU1-1	HKU1-2
Lowest EC50 (ng/mL)	6	18	10	5	7
Median EC50 (ng/mL)	14	41	26	10	16
Highest EC50 (ng/mL)	27	183	2935	713	977
Number of EC50 values in analysis	20	21	24	39	16

Note: Only EC50 values with full curve parameters were included in analysis.

**Table 3:** HCoV pseudovirus-neutralizing mAbs.

HCoV	mAb ID	ELISA EC50 (ng/mL)	Pseudovirus Neutralization IC50 (ng/mL)	Wild-Type Virus Neutralization IC50 (ng/mL)	Apparent K_D_ (M)	Kon (1/Ms)	Koff (1/s)	VH Germline	VL Germline
OC43	601_PACB2 (601_1A1)	13	994	438	1.6 × 10^−9^	9.0 × 10^4^	1.4 × 10^−4^	hu IGHV1-69	hu IGKV1-39
	601_PACB6	17	924	552	<1.0 × 10^−12^	2.1 × 10^5^	1.7 × 10^−7^	hu IGHV1-2	hu IGKV1-39
	601_PACB12	14	1129	940	2.7 × 10^−12^	7.0 × 10^4^	1.9 × 10^−7^	hu IGHV3-23	hu IGLV1-40
NL63	602_PACB5 (602_1B10)	18	0.7	51	3.0 × 10^−9^	5.1 × 10^5^	1.5 × 10^−3^	hu IGHV5-51	hu IGLV1-47
	602_PACB6	29	65	822	6.5 × 10^−12^	6.6 × 10^4^	4.3 × 10^−7^	hu IGHV3-23	hu IGKV1-39
	602_PACB11	41	5	174	3.5 × 10^−12^	1.3 × 10^5^	4.5 × 10^−7^	hu IGHV1-69	hu IGKV1-39
	602_PACB62 (602_1B1)	26	0.7	1183	5.1 × 10^−12^	9.7 × 10^4^	4.9 × 10^−7^	hu IGHV1-69	hu IGKV3-11
229E	603_PACB12	14	5	n.d.	6.5 × 10^−12^	6.7 × 10^4^	4.4 × 10^−7^	hu IGHV1-69	hu IGKV1-39
	603_PACB13	27	17	n.d.	5.9 × 10^−12^	8.4 × 10^4^	4.9 × 10^−7^	hu IGHV6-1	hu IGKV1-39
	603_PACB18	132	23	n.d.	1.0 × 10^−11^	3.8 × 10^4^	3.9 × 10^−7^	hu IGHV3-23	hu IGLV1-47
	603_PACB20	22	866	n.d.	3.0 × 10^−10^	2.5 × 10^6^	7.5 × 10^−4^	hu IGHV1-69	hu IGKV3-11
HKU1-1	604/5_PACB9 (604_1A12)	11	0.7	n.d.	4.0 × 10^−12^	4.0 × 10^6^	1.6 × 10^−5^	hu IGHV1-2	hu IGKV1-39
	604/5_PACB12 (604_1B3)	6	0.2	n.d.	1.7 × 10^−10^	1.7 × 10^6^	2.9 × 10^−4^	hu IGHV1-2	hu IGKV1-39
	604/5_PACB21	9	0.06	n.d.	<1.0 × 10^−12^	7.5 × 10^5^	<1.0 × 10^−7^	hu IGHV1-2	hu IGKV3-11
HKU1-2	604/5_PACB28 (605_1D10)	8	9	n.d.	9.9 × 10^−11^	3.2 × 10^6^	3.2 × 10^−4^	hu IGHV5-51	hu IGKV1-39
	604/5_PACB43 (605_1C4)	18	26	n.d.	1.3 × 10^−12^	3.8 × 10^5^	4.8 × 10^−7^	hu IGHV6-1	hu IGKV1-39
	604/5_PACB56	4	32	n.d.	5.6 × 10^−10^	4.2 × 10^5^	2.3 × 10^−4^	hu IGHV5-51	hu IGKV1-39

Note: Antibodies with an additional ID in parenthesis were identified from both traditional and NGS workflows.

**Table 4:** HCoV HKU1-1/1-2 pseudovirus cross-neutralizing mAbs.

HCoV	mAb ID	ELISA EC50 on HKU1-1 (ng/mL)	Pseudovirus Neutralization IC50 HKU1-1 (ng/mL)	Apparent K_D_ on HKU1-1 (M)	HKU1-1 Kon (1/Ms)	HKU1-1 Koff (1/s)	VH Germline	VL Germline
HKU1-1/HKU1-2	604/5_PACB45	5	409	<1.0 × 10^−12^	1.5 × 10^6^	<1.0 × 10^−7^	hu IGHV1-69	hu IGKV1D-16
	604/5_PACB80	8	388	<1.0 × 10^−12^	3.1 × 10^5^	<1.0 × 10^−7^	hu IGHV1-69	hu IGKV3-11
	mAb ID	ELISA EC50 on HKU1-2 (ng/mL)	Pseudovirus Neutralization IC50 HKU1-2 (ng/mL)	Apparent K_D_ on HKU1-2 (M)	HKU1-2 kon (1/Ms)	HKU1-2 koff (1/s)		
	604/5_PACB45	425	1708	2.1 × 10^−8^	1.5 × 10^7^	3.0 × 10^−1^		
	604/5_PACB80	977	2646	8.9 × 10^−9^	4.0 × 10^4^	3.5 × 10^−4^		

## Data Availability

The original contributions presented in this study are included in the article/Supplementary Materials. Further inquiries can be directed to the corresponding authors.

## Supplementary Materials

The following supporting information can be downloaded at: https://www.mdpi.com/article/10.3390/antib14020028/s1, Figure S1: ELISA confirmation of purified NGS-derived antibodies; Figure S2: BLI binding of neutralizing antibodies identified; Figure S3: Antibody heavy and light chains CDR3 germline analysis of expressed mAbs.

## References

[1] Corman V.M., Muth D., Niemeyer D., Drosten C. (2018). Hosts and Sources of Endemic Human Coronaviruses. Adv. Virus Res..

[2] Woo P.C.Y., Lau S.K.P., Huang Y., Yuen K.-Y. (2009). Coronavirus Diversity, Phylogeny and Interspecies Jumping. Exp. Biol. Med..

[3] Liu D., Chen C., Chen D., Zhu A., Li F., Zhuang Z., Mok C.K.P., Dai J., Li X., Jin Y. (2023). Mouse models susceptible to HCoV-229E and HCoV-NL63 and cross protection from challenge with SARS-CoV-2. Proc. Natl. Acad. Sci. USA.

[4] V’Kovski P., Kratzel A., Steiner S., Stalder H., Thiel V. (2021). Coronavirus biology and replication: Implications for SARS-CoV-2. Nat. Rev. Microbiol..

[5] McIntosh K., Dees J.H., Becker W.B., Kapikian A.Z., Chanock R.M. (1967). Recovery in tracheal organ cultures of novel viruses from patients with respiratory disease. Proc. Natl. Acad. Sci. USA.

[6] Hulswit R.J.G., Lang Y., Bakkers M.J.G., Li W., Li Z., Schouten A., Ophorst B., Van Kuppeveld F.J.M., Boons G.-J., Bosch B.-J. (2019). Human coronaviruses OC43 and HKU1 bind to 9-*O*-acetylated sialic acids via a conserved receptor-binding site in spike protein domain A. Proc. Natl. Acad. Sci. USA.

[7] Salvatori G., Luberto L., Maffei M., Aurisicchio L., Roscilli G., Palombo F., Marra E. (2020). SARS-CoV-2 SPIKE PROTEIN: An optimal immunological target for vaccines. J. Transl. Med..

[8] Smith G.P. (1985). Filamentous Fusion Phage: Novel Expression Vectors That Display Cloned Antigens on the Virion Surface. Science.

[9] Scott J.K., Smith G.P. (1990). Searching for Peptide Ligands with an Epitope Library. Science.

[10] Breitling F., Dübel S., Seehaus T., Klewinghaus I., Little M. (1991). A surface expression vector for antibody screening. Gene.

[11] Barbas C.F., Kang A.S., Lerner R.A., Benkovic S.J. (1991). Assembly of combinatorial antibody libraries on phage surfaces: The gene III site. Proc. Natl. Acad. Sci. USA.

[12] McCafferty J., Griffiths A.D., Winter G., Chiswell D.J. (1990). Phage antibodies: Filamentous phage displaying antibody variable domains. Nature.

[13] Zhang Y. (2023). Evolution of phage display libraries for therapeutic antibody discovery. mAbs.

[14] Tiller T., Schuster I., Deppe D., Siegers K., Strohner R., Herrmann T., Berenguer M., Poujol D., Stehle J., Stark Y. (2013). A fully synthetic human Fab antibody library based on fixed VH/VL framework pairings with favorable biophysical properties. mAbs.

[15] Hust M., Lim T.S., Phage Display (2018). Methods in Molecular Biology.

[16] Ravn U., Didelot G., Venet S., Ng K.T., Gueneau F., Rousseau F., Calloud S., Kosco-Vilbois M., Fischer N. (2013). Deep sequencing of phage display libraries to support antibody discovery. Methods.

[17] Deschaght P., Vintém A.P., Logghe M., Conde M., Felix D., Mensink R., Gonçalves J., Audiens J., Bruynooghe Y., Figueiredo R. (2017). Large Diversity of Functional Nanobodies from a Camelid Immune Library Revealed by an Alternative Analysis of Next-Generation Sequencing Data. Front. Immunol..

[18] Turner K.B., Naciri J., Liu J.L., Anderson G.P., Goldman E.R., Zabetakis D. (2016). Next-Generation Sequencing of a Single Domain Antibody Repertoire Reveals Quality of Phage Display Selected Candidates. PLoS ONE.

[19] Nakada-Masuta T., Takeda H., Uchida K. (2023). Novel Approach for Obtaining Variable Domain of New Antigen Receptor with Different Physicochemical Properties from Japanese Topeshark (*Hemitriakis japanica*). Mar. Drugs.

[20] Lövgren J., Pursiheimo J.P., Pyykkö M., Salmi J., Lamminmäki U. (2016). Next generation sequencing of all variable loops of synthetic single framework scFv-Application in anti-HDL antibody selections. New Biotechnol..

[21] Glanville J., D’Angelo S., Khan T., Reddy S., Naranjo L., Ferrara F., Bradbury A. (2015). Deep sequencing in library selection projects: What insight does it bring?. Curr. Opin. Struct. Biol..

[22] Dekosky B.J., Kojima T., Rodin A., Charab W., Ippolito G.C., Ellington A.D., Georgiou G. (2015). In-depth determination and analysis of the human paired heavy- and light-chain antibody repertoire. Nat. Med..

[23] Rhoads A., Au K.F. (2015). PacBio Sequencing and its Applications. Genom. Proteom. Bioinform..

[24] Hemadou A., Giudicelli V., Smith M.L., Lefranc M.-P., Duroux P., Kossida S., Heiner C., Hepler N.L., Kuijpers J., Groppi A. (2017). Pacific Biosciences Sequencing and IMGT/HighV-QUEST Analysis of Full-Length Single Chain Fragment Variable from an In Vivo Selected Phage-Display Combinatorial Library. Front. Immunol..

[25] Nannini F., Senicar L., Parekh F., Kong K.J., Kinna A., Bughda R., Sillibourne J., Hu X., Ma B., Bai Y. (2021). Combining phage display with SMRTbell next-generation sequencing for the rapid discovery of functional scFv fragments. mAbs.

[26] Han S.Y., Antoine A., Howard D., Chang B., Chang W.S., Slein M., Deikus G., Kossida S., Duroux P., Lefranc M.P. (2018). Coupling of Single Molecule, Long Read Sequencing with IMGT/HighV-QUEST Analysis Expedites Identification of SIV gp140-Specific Antibodies from scFv Phage Display Libraries. Front. Immunol..

[27] Prassler J., Thiel S., Pracht C., Polzer A., Peters S., Bauer M., Nörenberg S., Stark Y., Kölln J., Popp A. (2011). HuCAL PLATINUM, a Synthetic Fab Library Optimized for Sequence Diversity and Superior Performance in Mammalian Expression Systems. J. Mol. Biol..

[28] Chen Z., Zhang L., Tang A., Callahan C., Pristatsky P., Swoyer R., Cejas P., Nahas D., Galli J., Cosmi S. (2016). Discovery and Characterization of Phage Display-Derived Human Monoclonal Antibodies against RSV F Glycoprotein. PLoS ONE.

[29] Chen F., Tzarum N., Wilson I.A., Law M. (2019). VH1-69 antiviral broadly neutralizing antibodies: Genetics, structures, and relevance to rational vaccine design. Curr. Opin. Virol..

[30] Weber T., Potthoff J., Bizu S., Labuhn M., Dold L., Schoofs T., Horning M., Ercanoglu M.S., Kreer C., Gieselmann L. (2022). Analysis of antibodies from HCV elite neutralizers identifies genetic determinants of broad neutralization. Immunity.

[31] Wang Y., Zhao Y., Bollas A., Wang Y., Au K.F. (2021). Nanopore sequencing technology, bioinformatics and applications. Nat. Biotechnol..

[32] Garcia-Calvo E., García-García A., Rodríguez S., Farrais S., Martín R., García T. (2022). Construction of a Fab Library Merging Chains from Semisynthetic and Immune Origin, Suitable for Developing New Tools for Gluten Immunodetection in Food. Foods.

