# Supplementary material for: Discovery of Antibodies Against Endemic Coronaviruses with NGS-Based Human Fab Phage Display Platform
Source: Antibodies (Basel). 2025 Mar 27;14(2):28. doi: 10.3390/antib14020028 (PMC12015876; doi:10.3390/antib14020028)
Supplement: Supplementary file 1 [file antibodies-14-00028-s001.zip › antibodies-3515817-supplementary.pdf]

Supplementary Materials:

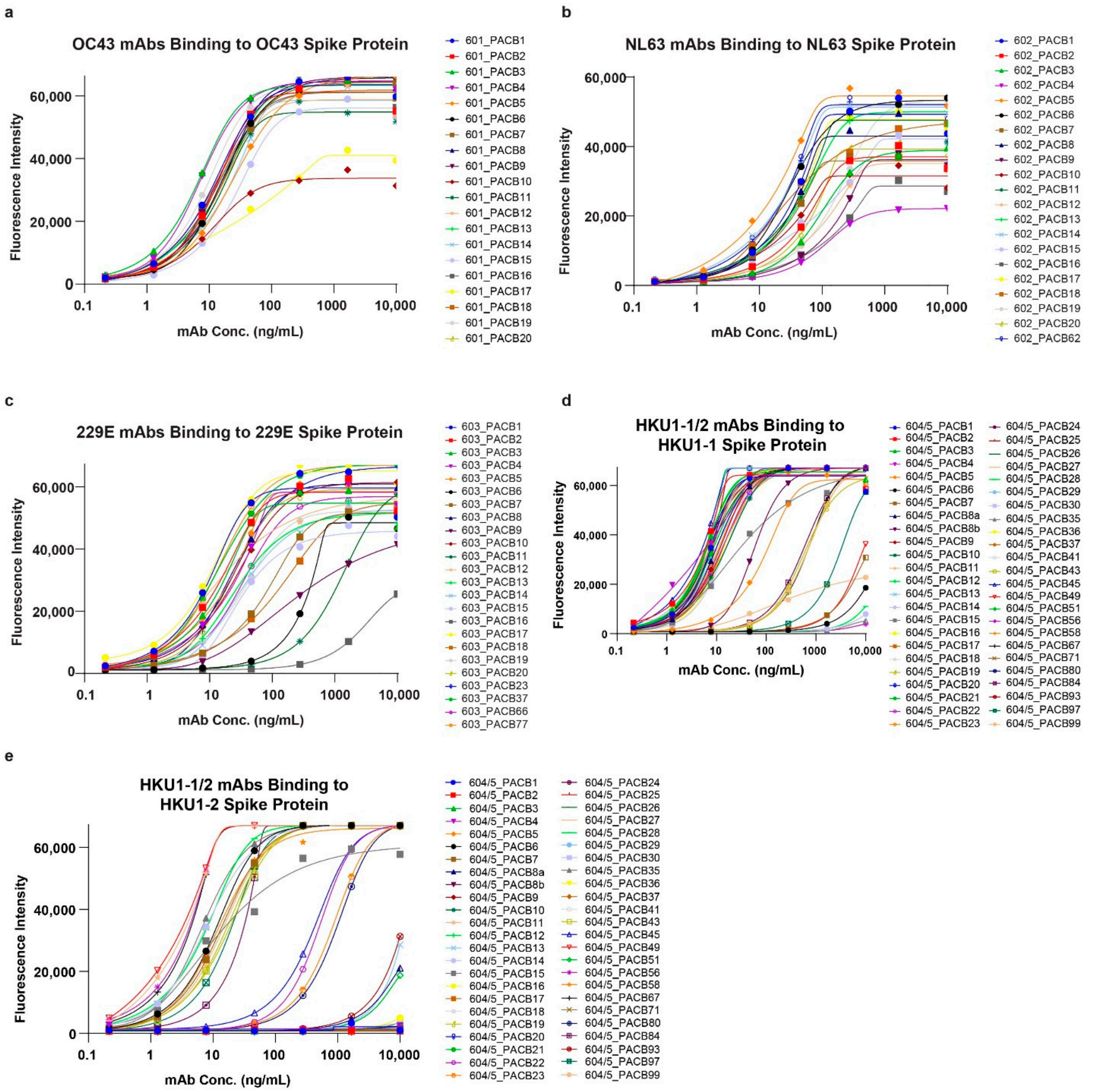

Figure S1. ELISA confirmation of purified NGS-derived antibodies. Antigens were coated at 1 ug/mL overnight. Antibodies were serially titrated from 10 ug/mL with 6-fold dilutions. The numbering in the antibody name reflected their respective frequency ranking from NGS. Antibodies from each set of cross-panning (example: NL63 and OC43) were tested by ELISA binding on both antigens to show antigen-specificity. Results (not shown) were negative for NL63 antibodies binding to OC43 antigen and vice versa. In this manner NL63 antibodies served as negative controls

for OC43 antibodies in testing against ELISA binding to OC43 antigen. Same protocol applied to 229E and HKU antibodies.

Biolayer interferometry binding affinity measurements

Biolayer interferometry (BLI) assays were performed using default kinetics measurement protocol on an Octet Red 96 instrument (Sartorius, Bohemia, NY, USA). A baseline was established before and after the loading of biotin-labeled spike proteins by immersing them into wells containing 1× Octet kinetics buffer (Sartorius). Spike proteins (3 µg/mL) conjugated with EZ-Link NHS-peg4-biotin (Thermo Fisher Scientific) were immobilized onto streptavidin biosensors (Sartorius). Finally, the antibodies at various concentrations starting from 100 nM in 1× Octet kinetics buffer were associated for 5 minutes and dissociated for 5 minutes in wells with 1× Octet kinetics buffer. After each run, results were analyzed with instrument software using global fit and a 2:1 heterogeneous model. Data points were graphed using GraphPad Prism (version 10.2.2) for clarity.

Figure S2. BLI binding of neutralizing antibodies identified. Biotinylated spike proteins were captured on streptavidin sensor. Serially diluted antibodies were measured for association and dissociation binding rates. Data were analyzed with global fit and 2:1 heterogeneous model. .

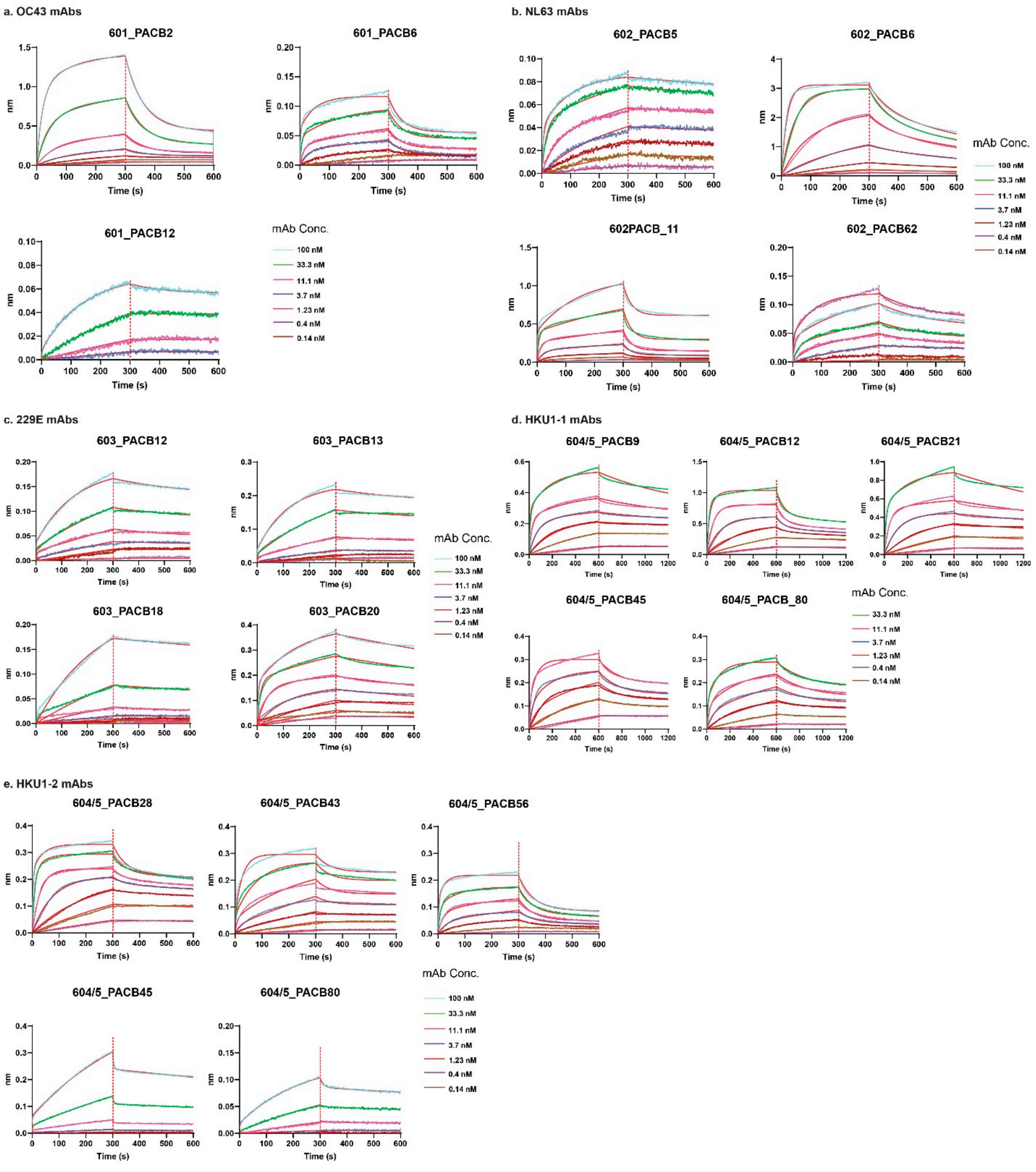

a. OC43 mAbs
601_PACB2
601_PACB6
601_PACB12
mAb Conc.
100 nM
33.3 nM
11.1 nM
3.7 nM
1.23 nM
0.4 nM
0.14 nM
b. NL63 mAbs
602_PACB5
602_PACB6
602PACB_11
602_PACB62
c. 229E mAbs
603_PACB12
603_PACB13
603_PACB18
603_PACB20
d. HKU1-1 mAbs
604/5_PACB9
604/5_PACB12
604/5_PACB21
604/5_PACB45
604/5_PACB_80
e. HKU1-2 mAbs
604/5_PACB28
604/5_PACB43
604/5_PACB56
604/5_PACB45
604/5_PACB80
nm
Time (s)

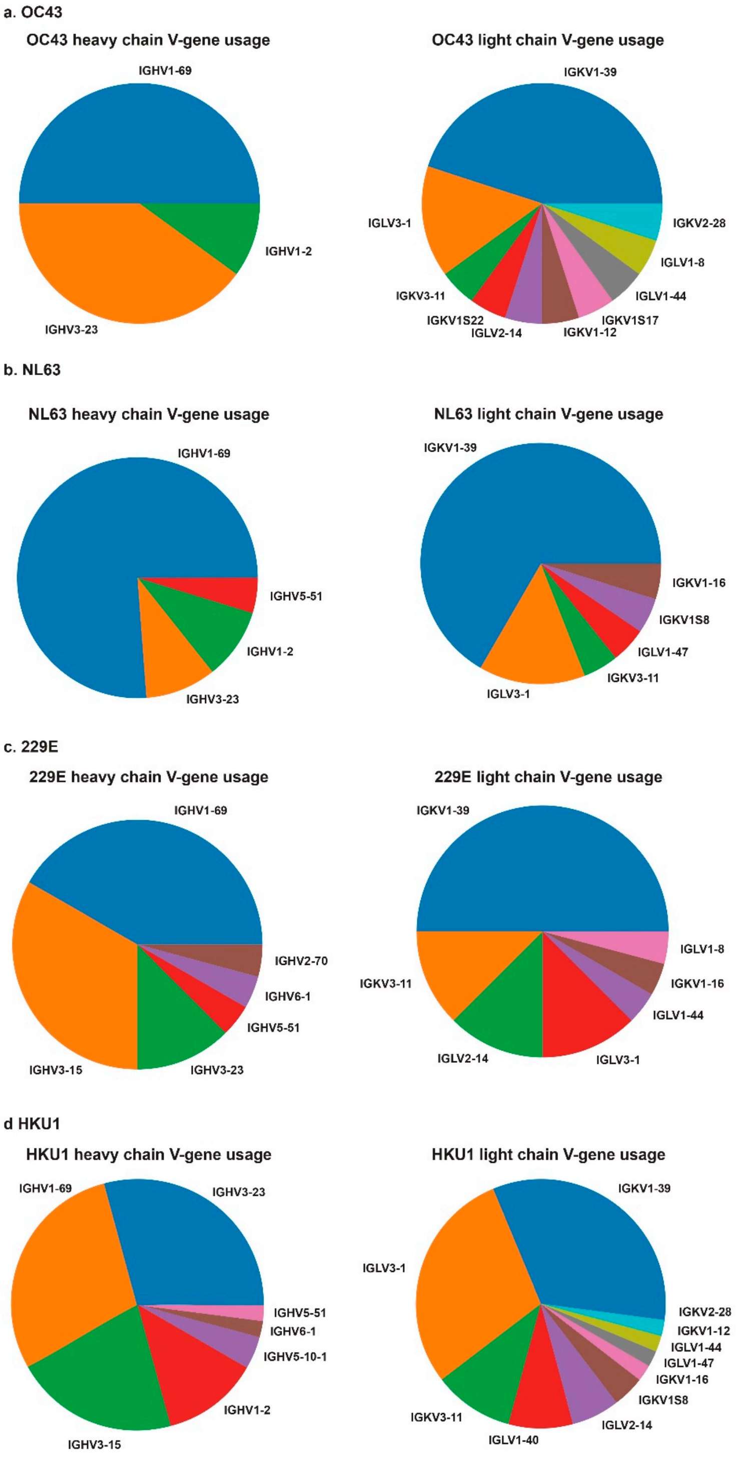

Figure S3. Antibody heavy and light chains CDR3 (Kabat antibody numbering) germline analysis of expressed mAbs with high frequency by NGS against spike protein of endemic coronaviruses. Each pie chart is composed of the percentage of germline usage of NGS-derived antibodies.
